# Cellular signaling pathway of Shiga toxin-induced ATP release

**DOI:** 10.3389/fcimb.2026.1705239

**Published:** 2026-02-23

**Authors:** Karl Johansson, Ida Arvidsson, Markus Wendler, Ann-Charlotte Kristoffersson, Diana Karpman

**Affiliations:** Department of Pediatrics, Clinical Sciences Lund, Lund University, Lund, Sweden

**Keywords:** ATP, G protein, globotriaosylceramide, inositol 1,4,5-triphosphate receptor, phosphatidylinositol 4,5-bisphosphate, Shiga toxin

## Abstract

**Background:**

Shiga toxin (Stx) is the main virulence factor of enterohemorrhagic *Escherichia coli*, a food-borne pathogen that colonizes the intestine causing gastroenteritis and, in severe cases, hemolytic uremic syndrome. Stx was shown to induce ATP release *in vivo* and *in vitro* and blockade of purinergic P2X receptors inhibited its cytotoxicity. Here we investigated the intracellular signaling events preceding ATP release.

**Methods:**

Inhibitors included pertussis toxin, wortmannin, manoalide, 2-aminoethoxydiphenylborate (2-APB), BAPTA-AM and Ca^2+^-free medium. The inositol 1,4,5-triphosphate receptor (IP_3_R) was silenced. Stx-induced apoptosis was detected by caspase 3/7 activation. BALB/c mice were injected with Stx2 i.p. Certain mice were pretreated with alpelisib (1 h before and 24 h after Stx2). Kidneys collected after 4 days were stained for phosphatidylinositol 4,5-bisphosphate (PIP2).

**Results:**

Stx1-mediated ATP release in HeLa cells was blocked by pertussis toxin affecting the Gi/o family of G-protein coupled receptors. ATP release was also abrogated by wortmannin, an inhibitor of phosphoinositide 3-kinase (PI3K), by manoalide, inhibiting phospholipase C, by 2-APB inhibiting IP_3_R, and by reduction of intracellular calcium (BAPTA-AM) and extracellular calcium (Ca^2+^-free medium). Blocking or silencing the IP_3_R protected HeLa cells from Stx1-induced apoptosis. Likewise, blocking the IP_3_R reduced Stx2-induced apoptosis. Stx2-challenged mice had distinct PIP2 glomerular staining that decreased in the presence of the PI3K inhibitor alpelisib.

**Conclusion:**

Stx interaction with HeLa cells initiates a signaling pathway involving membrane G protein, PI3K, phospholipase C and IP_3_R, ultimately leading to ATP release and promoting cytotoxic effects. The PI3K inhibitor alpelisib altered PIP2 expression in Stx2-challenged mice.

## Introduction

Shiga toxin (Stx) is a bacterial toxin that is produced by Shiga toxin-producing *Escherichia coli* (STEC). The strains are non-invasive, colonizing the intestine. A subset of STEC strains capable of causing hemorrhagic colitis are termed enterohemorrhagic *Escherichia coli* (EHEC). Certain patients develop the severe complication hemolytic uremic syndrome, characterized by hemolytic anemia, thrombocytopenia and acute renal failure with high morbidity and the risk of death (Tarr et al., 2005). The toxin is an AB5 toxin that consists of a pentameric B-subunit, which mediates binding to its receptor, and an A-subunit that is enzymatically active (Endo et al., 1988; Fraser et al., 1994). The toxin binds to globotriaosylceramide (Gb3), a glycosphingolipid that is present in the plasma membrane of certain cells (Gallegos et al., 2012). After binding, the holotoxin undergoes retrograde transport via early endosomes and the Golgi apparatus before reaching the endoplasmic reticulum. In the endoplasmic reticulum a fragment of the A-subunit is cleaved by furin and then released into the cytosol, where it mediates depurination of a specific adenine base in the 28S ribosomal RNA (Endo et al., 1988; Garred et al., 1995) thereby inhibiting protein translation and leading to cell death. There are two main subtypes of Stx, Stx1 and Stx2. Both utilize this mechanism to induce target organ cell injury (Sandvig and van Deurs, 1996).

Stx binding to Gb3 on the cell surface has been shown to cause cellular activation, as demonstrated by elevated intracellular calcium levels as well as phospholipase C (PLC) activation (Liu et al., 2011; Klokk et al., 2016). Our group has previously shown that Stx1 induced ATP release from cells and Stx2 also exhibited a similar tendency (Johansson et al., 2019). Moreover, the effect of Stx2 on ATP release was demonstrated *in vivo* in a mouse model (Johansson et al., 2019). The released ATP signals via purinergic P2X receptors and thereby contributes to calcium influx into the cell and the damaging effects of Stx such as inhibition of protein synthesis, decreased cell viability, increased apoptosis, and shedding of extracellular vesicles. By blocking P2X receptors we could show that ATP plays a role in Stx-mediated apoptosis *in vitro* and shedding of extracellular vesicles both *in vitro* and *in vivo* (Johansson et al., 2019). Vesicle shedding contributes to the development of severe organ damage as these vesicles transport the toxin from the gut to the kidney (Ståhl et al., 2015). Apyrase is an enzyme that cleaves ATP to ADP and AMP, and it was shown to have multiple protective effects in our mouse model of EHEC infection (Arvidsson et al., 2022).

The intracellular content of ATP is considerably higher than the extracellular (Burnstock, 2007). ATP is released to the extracellular space by leakage from dying and damaged cells and it is also released from viable cells during hypoxia, shear stress, hypotonia, deformation and upon stimulation (Boudreault and Grygorczyk, 2004; Burnstock, 2007). ATP release involves vesicular release, demonstrated in nerve cells and inflammatory cells (Burnstock, 2007; Dosch et al., 2018), ATP-binding cassette (ABC) transporters as well as passage through pore formations generated by connexin or pannexin hemichannels (Lohman et al., 2012; Dosch et al., 2018). The stimulant associated with ATP release may vary but generally seems to be tightly linked to an increase in intracellular calcium levels (Boudreault and Grygorczyk, 2004).

In this study we aimed to investigate the cellular mechanisms involved in Stx-mediated ATP release from HeLa cells. To this end we used an inactivator of the Gi/o alpha subunit of G-protein coupled receptors (GPCRs) and various inhibitors of intracellular enzymes and receptors, blocking phosphoinositide 3-kinase (PI3K), PLC, inositol 1,4,5-triphosphate receptor (IP_3_R) and affecting intracellular calcium levels. Activation of the PI3K-IP_3_R pathway was further investigated by silencing of IP_3_R and by phosphatidylinositol 4,5-bisphosphate (PIP2) staining of kidney tissue in Stx2-challenged mice. By addressing the mechanism in which Stx mediates ATP release, associated with toxin-mediated cellular injury, we hope to define suitable targets for inhibiting the cellular effects of Stx.

## Methods

### Cell culture

HeLa cells (a kind gift from Ludger Johannes, Institute Curie, Paris) were cultured in Dulbecco’s Modified Eagle Medium (DMEM) supplemented with 10% fetal calf serum and 1% penicillin-streptomycin (all from Gibco, Waltham, MA) in 5% CO_2_ at 37°C.

### Shiga toxin

Stx1a and Stx2a were acquired from the Division of Geographic Medicine and Infectious Disease, Tufts Medical Center, Boston MA. Lipopolysaccharide content was assessed using the Limulus Amebocyte Lysate assay (Thermo Fisher Scientific, Rockford, IL) and found to be 2.3 ng/mg Stx1 and 69 ng/mg Stx2.

### Cellular ATP release assay

HeLa cells were seeded out in transparent 96-well plates (10,000 cells per well, Corning Inc, Corning, NY) 24 h before the start of the experiment. The outer wells of the plate were not used. Cells were washed twice and incubated in Hank’s Balanced Salt Solution (HBSS), with or without Ca^2+^ (1.26 mM), supplemented with 20 mM 4-(2-hydroxytheyl)-1-piperazineethanesulfonic acid (HEPES) (both from Thermo Fisher Scientific) with or without the inhibitors listed in **Table 1**, or with their corresponding vehicle, at the same volume, for 30 min or, in the case of pertussis toxin, for 16-20 h, as the latter undergoes retrograde transport before it can bind to its target (Katada et al., 1983). Stx1 (final concentration 1 μg/mL) diluted in HBSS, or HBSS alone (negative control), was then added to the cells. Distilled water (50% dilution) was used as the positive control, as a drop in osmolality has been shown to induce ATP release (Taylor et al., 1998). After 5 min incubation the supernatant was collected, without disturbing the cells. The supernatant was added to white 96-well plates (Corning) together with d-luciferin (1.2 mM) and luciferase (65 nM, both from Thermo Fisher Scientific) diluted in HBSS, and the ATP content was determined by luminescence measurement at 1s integration time in a Glomax Discover System (Promega, Madison, WI). Experiments in which Stx1 did not increase ATP release (8/43) were excluded from evaluation with any inhibitors.

**Table 1:** Cellular pathway inhibitors used in this study.

Inhibitor	Concentration	Pathway affected	Reference
Pertussis toxin^a,b^	1.1 nM	Gα_i_ inhibitor	(Keen et al., 2022)
Wortmannin^c,d^	1 μM	Phosphoinositide 3-kinase inhibitor	(Bernier et al., 2008)
Manoalide^d,e^	100 nM	Phospholipase C inhibitor	(Yu et al., 2017)
2-APB^c,d^	100 μM	Inositol 1,4,5-triphosphate receptor inhibitor	(Bishara et al., 2002)
BAPTA-A^d,f^	20 μM	Chelates free cytosolic Ca^2+^	(Huang et al., 2013)

^a^Tocris, ^b^dissolved in H_2_O, ^c^Sigma-Aldrich, ^d^dissolved in DMSO (constituting 0.1% of the final volume), ^e^Santa Cruz, ^f^Thermo Fisher Scientific; For each inhibitor at least three different concentrations were tested. 2-APB: 2-aminoethoxydiphenylborate.

### Silencing of inositol 1,4,5-triphosphate receptor expression

IP_3_R mRNA in HeLa cells was silenced by small interfering RNA (siRNA). Cells were transfected with a pool of three different siRNA targeting IP_3_R mRNA (siIP_3_RI, IP_3_RII and IP_3_RIII) or with a non-targeting control siRNA (siCtrl) using the Transfection Reagent according to the manufacturer’s instructions (all from Santa Cruz Biotechnology, Santa Cruz, CA). Experiments were carried out 72 h post transfection. Immunoblotting was performed to confirm protein reduction (IP_3_R antibody from Abcam Cambridge, UK, catalogue #108517) as presented in **Supplementary Figure 1**. Equal protein concentrations were determined by GAPDH (Abcam, AB125247).

### Caspase activity assay

ATP signaling was previously shown to be involved in Stx1-mediated apoptosis, as measured by caspase 3/7 activity (Johansson et al., 2019). HeLa cells were seeded out in Ibidi 8-well chamber slides (30,000 cells per well) 24 h before the start of the experiment. The cells were washed twice in FluoroBrite (Thermo Fisher Scientific) and incubated with 2-APB (1, 10 or 100 μM), wortmannin (0.1, 1 or 10 μM), manoalide (10, 100 or 1000 μM) or DMSO for 30 min, followed by Stx1 or Stx2 (7 ng/mL). In a separate experiment, HeLa cells that had been treated with siRNA targeting IP_3_R or with control siRNA (siCtrl) were incubated with Stx1 (7 ng/mL) or PBS control. Stx1 and Stx2 at the concentration used for ATP release (1 μg/mL) caused cell detachment at 24 h and therefore a lower concentration was chosen for the caspase 3/7 activity assay, as previously described (Johansson et al., 2019). Stx1 at the lower concentration of 7 ng/mL was previously shown to induce apoptosis after 24 h with negligible cell detachment. After 24 h CellEvent Caspase 3/7 reagent (2 μM) and NucBlue Live ReadyProbe (both from Thermo Fisher Scientific) were added for 30 min. Cells were imaged using a Nikon Eclipse Ti-E microscope equipped with Hamamatsu Flash 4 camera using NIS Elements AR software v.5.11.01 (Nikon Instruments Inc., Tokyo, Japan) and analyzed using ImageJ Fiji. The number of nuclei, the area of fluorescence and mean intensity of CellEvent caspase 3/7 fluorescence within an area of 2720 x 2720 μm were measured. The area of fluorescence was multiplied by the mean intensity of fluorescence and divided by the number of nuclei to attain fluorescence per cell. Cells incubated with Stx1 or Stx2 alone (without inhibitors) were defined as having 100% caspase 3/7 activation.

### BALB/c mice injected with Stx2

BALB/c wild-type mice were bred at the animal facilities of Lund University under standard laboratory conditions. Both male and female mice, aged 8–12 weeks, were used. Mice received intraperitoneal (i.p.) injections of either the PIK3 inhibitor alpelisib (5 mg/kg, MedChemExpress, NJ), its vehicle (DMSO), or PBS 1 h before challenge with Stx2 (142 ng/kg) or PBS i.p. Treatments were repeated 24 h after Stx2 injection. All mice were sacrificed by cervical dislocation under 4% isoflurane inhalation (Piramal Critical Care, Andhra Pradesh, India) 4 days after Stx2 injection. Kidneys were harvested, fixed in 4% paraformaldehyde (PFA, Histolab Products AB, Askim, Sweden), and embedded in paraffin for histological analysis. Animal experiments were approved by the regional Animal Ethics Committee (#17452-20) and performed in accordance with regulations of the Swedish Board of Agriculture and the European Directive on the protection of animals used for scientific purposes.

### Immunofluorescence staining of PIP2 in mouse kidneys

Mouse renal tissue was deparaffinized and stained as previously described (Lopatko Fagerström et al., 2019). Sections were blocked with goat (Fab) anti-mouse IgG 10 μg/mL (Abcam ab6668) in 5% bovine serum albumin (BSA, Sigma-Aldrich) for 1h at RT followed by incubation with mouse monoclonal IgM anti-PIP2 20 μg/mL (Abcam, AB11039) overnight at 4°C. Incubation with secondary antibody goat anti-mouse alexa flour 488 (Invitrogen ab2534062) 1:400 for 1h at RT was followed by staining with NucBlue (Thermo Fisher Scientific) according to the manufacturer’s instructions and mounted using antifade fluorescence mounting medium (Abcam). The sections were analyzed with the Nikon Eclipse Ti-E microscope. Sections were assessed for the staining pattern in an entire kidney section and specifically for positive staining in glomeruli, and the number of positively stained glomeruli was divided by the total number of glomeruli in the section.

### Statistical analysis

The Wilcoxon signed-rank test was used for comparison of paired data and the Mann-Whitney U test was used for comparisons between two groups. The Kruskal-Wallis multiple-comparison test, followed by Dunn’s procedure, was used for comparisons between multiple groups. All tests were performed using GraphPad Prism version 9.0.0 (86) for Mac (GraphPad Software, San Diego, CA).

## Results

### Stx1 induces ATP release by G-protein activation

**Figure 1:**
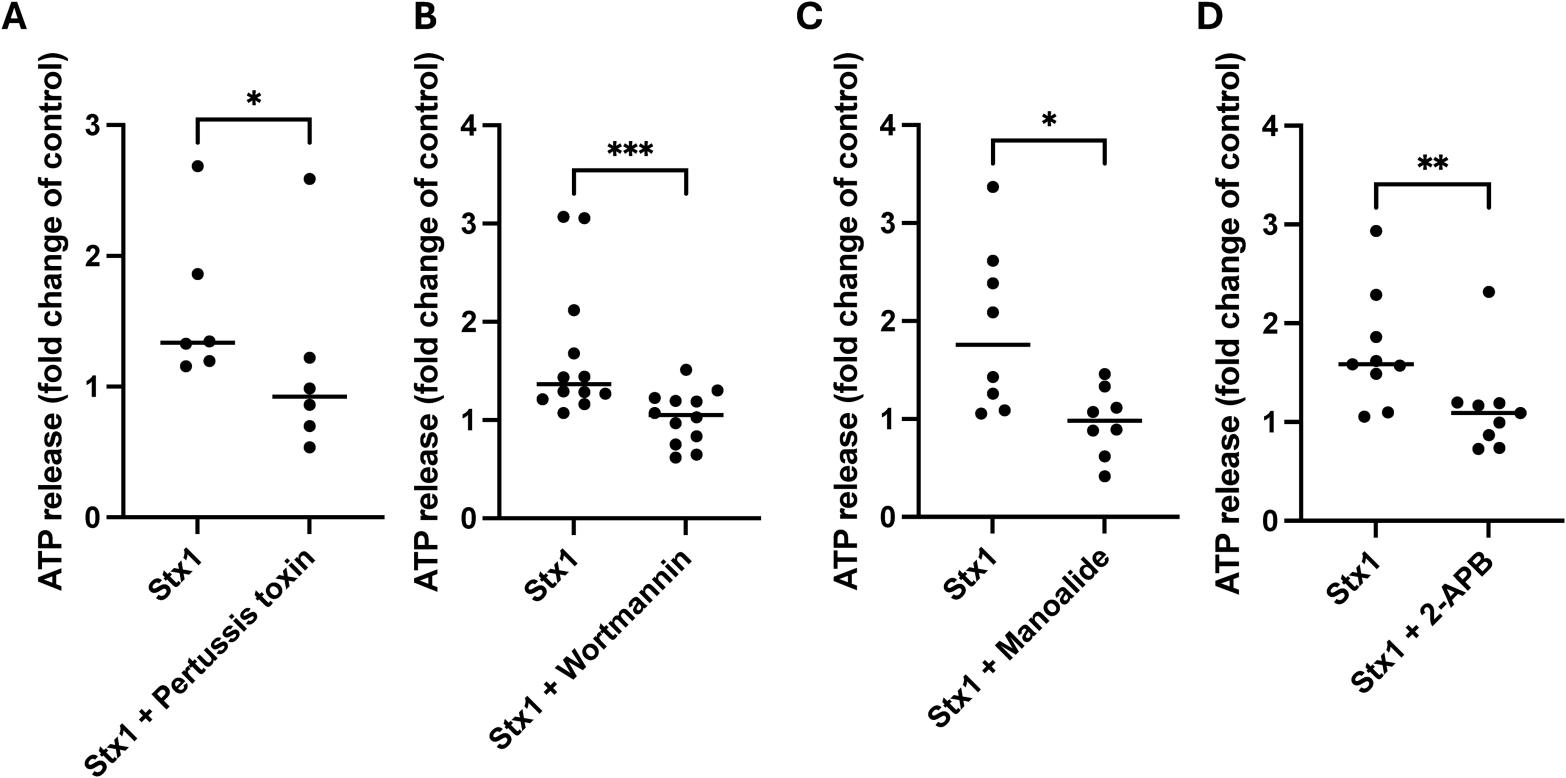
Shiga toxin 1-induced ATP release is inhibited by Pertussis toxin, wortmannin, manoalide and 2-APB HeLa cells were incubated with inhibitors of cellular signaling pathways. **(A)** HeLa cells were incubated with Pertussis toxin (Gi/o-protein blocker) or with PBS vehicle (n=6) followed by Shiga toxin 1 (Stx1). ATP release was significantly lower in cells treated with Pertussis toxin. **(B)** HeLa cells were treated with Wortmannin (phosphoinositide 3-kinase inhibitor) or DMSO (vehicle) before addition of Stx1 (n=12). A significantly lower release of ATP was observed from cells treated with wortmannin compared to untreated cells. **(C)** HeLa cells were treated with manoalide (phospholipase C inhibitor) or DMSO (vehicle) followed by stimulation with Stx1 (n=8). A significantly lower ATP release was observed in Stx1-stimulated manoalide-treated cells compared to untreated cells (DMSO). **(D)** HeLa cells were treated with 2-APB (IP3 receptor inhibitor) or DMSO (vehicle) followed by stimulation with Stx1 (n=9). A significantly lower ATP release was observed in Stx1-stimulated 2-APB-treated cells compared to untreated cells (DMSO). Samples were normalized to 1 based on the vehicle samples. Source data are presented in **Supplementary Figure 2**. Each data point represents a mean of four technical repeats from one experiment. Median ATP release is denoted by the bar. *P <0.05, **P <0.01, ***P <0.001. Wilcoxon signed-rank test in which data from the same experiment were paired.

Stx1-stimulates ATP release from HeLa cells (Johansson et al., 2019). Pertussis toxin blocks the activation of the α-subunit of Gi/o-proteins associated with GPCRs (Katada et al., 1983). HeLa cells treated with Pertussis toxin exhibited significantly decreased ATP release after stimulation with Stx1 compared to untreated cells (**Figure 1A**, for source data and luminescence values associated with **Figure 1** see **Supplementary Figure 2A**).

### Stx1 induces ATP release via phosphoinositide 3-kinase signaling

Wortmannin, an inhibitor of PI3K, blocks PIP2 phosphorylation to phosphatidylinositol 3,4,5-trisphosphate (PIP3) by PI3K (Huang et al., 2011). Wortmannin significantly reduced ATP release in Stx1-stimulated HeLa cells, compared to untreated cells (**Figure 1B**, for source data see **Supplementary Figure 2B**). These results suggest that PIP2 signaling may be involved in Stx1-mediated ATP release.

### Stx1-induced ATP release involves phospholipase C and the inositol 1,4,5-triphosphate receptor

HeLa cells treated with manoalide, an inhibitor that blocks most isoforms of PLC at higher concentrations (Bennett et al., 1987), exhibited significantly reduced Stx1-induced ATP release compared to control cells (**Figure 1C**). Stx1-stimulated HeLa cells treated with 2-APB, an inhibitor of the IP_3_R (Bishara et al., 2002), also had a significantly lower amount of ATP in the cell supernatant compared to untreated cells (**Figure 1D**). These results indicate that Stx1 signaling, and secondary ATP release, involve PLC and IP_3_ binding to the IP_3_-calcium channel receptor. For source data see **Supplementary Figures 2C, D**.

### Shiga toxin induced caspase activation involves the IP_3_ receptor

**Figure 2:**
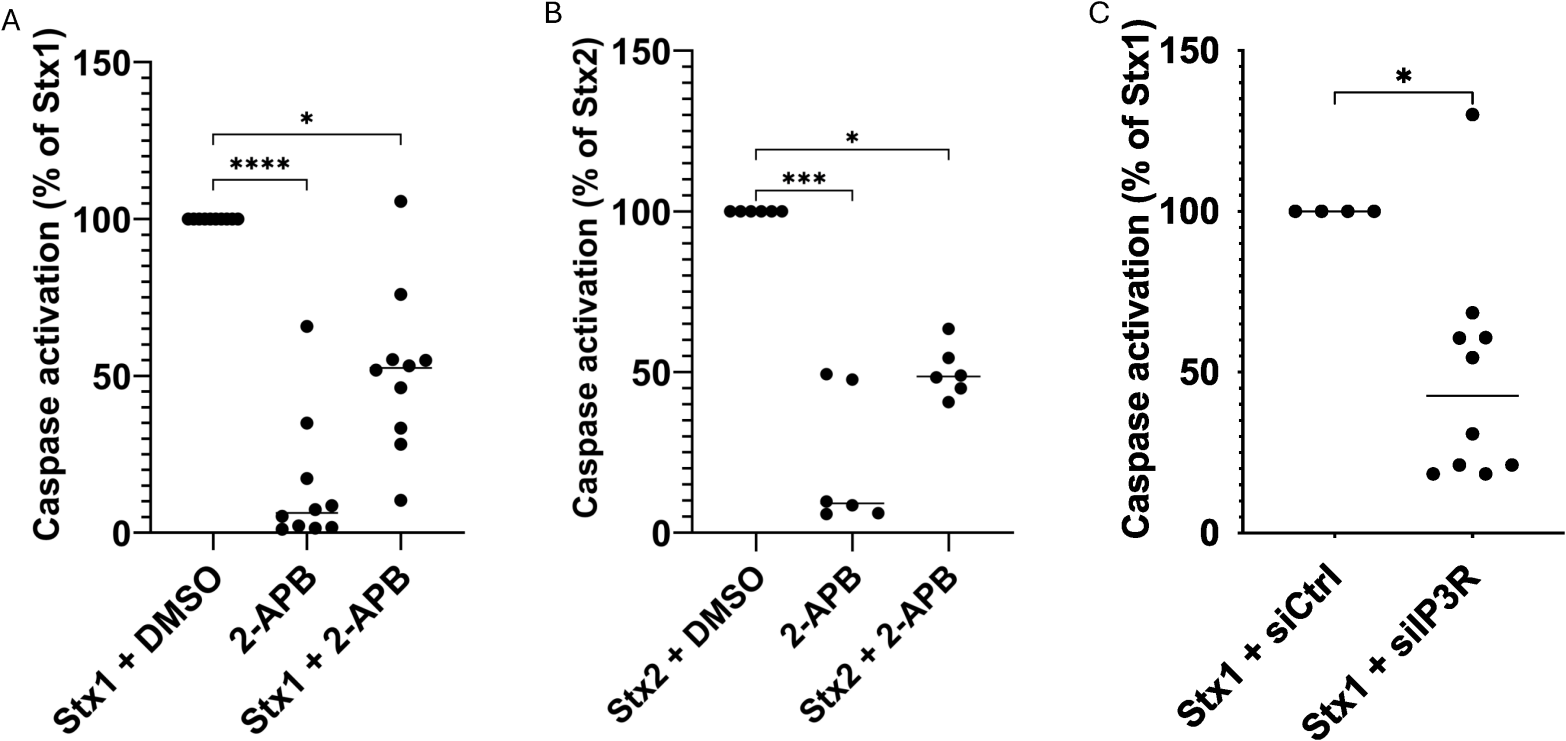
The IP_3_ receptor is involved in Shiga toxin 1-mediated caspase 3/7 activation HeLa cells were treated with 2-APB or DMSO followed by Shiga toxin 1 (Stx1) or Shiga toxin 2 (Stx2) for 24 h and analyzed for apoptosis as determined by caspase 3/7 activation. **(A)** Stx1-stimulated cells (with DMSO vehicle) had significantly more caspase 3/7 activation compared to cells treated with 2-APB (n=10 independent experiments). Stx1 data was defined as 100% caspase 3/7 activation based on values in the source data (**Supplementary Figure 3A**). Each data point was acquired from all cells in 16 separate images from one well. **(B)** Stx2-stimulated cells (with DMSO vehicle) exhibited significantly more caspase 3/7 activation compared to cells treated with 2-APB. Each data point was acquired from all cells in 16 separate images from one sample. Stx2 data was defined as 100% caspase 3/7 activation based on values in the source data (**Supplementary Figure 3B**). **(C)** HeLa cells were treated with siRNA targeting IP_3_R (siIP_3_R) or a control siRNA (siCtrl) for 72 h followed by Stx1 for 24 h and analyzed for Stx1-induced caspase 3/7 activation. Cells treated with siIP_3_R had significantly less caspase 3/7 activation compared to cells treated with siCtrl. Each data point was acquired from all cells in 16 separate images from one sample. Stx1 + siCtrl data was defined as 100% caspase 3/7 activation based on values in the source data (**Supplementary Figure 3C**) *P <0.05, ***P<0.001, ****P <0.0001, Kruskal-Wallis test, followed by Dunn’s procedure in **(A, B)**, Mann-Whitney U test in **(C)**.

Purinergic signaling is involved in Stx1-mediated apoptosis (Johansson et al., 2019). As wortmannin, manoalide and 2-APB decreased Stx1-induced ATP release from HeLa cells, we investigated if these inhibitors protected HeLa cells from Stx1-induced apoptosis. A significant caspase activation was seen in almost all cells that were incubated with Stx1. 2-APB-treated cells stimulated with Stx1 also exhibited caspase activation but to a significantly lesser extent (**Figure 2A**, for source data see **Supplementary Figure 3A**). Similar results were obtained using Stx2 and 2-APB-treated cells (**Figure 2B**, for source data see **Supplementary Figure 3B**). 2-APB alone did not induce considerable apoptosis compared to untreated cells.

Neither wortmannin- nor manoalide-treated cells stimulated with Stx1 were protected from caspase activation (**Supplementary Figure 4**). This indicates that the target receptor of 2-APB, IP_3_R, is involved in the induction of Stx1- and Stx2-mediated apoptosis in HeLa cells.

HeLa cells in which IP_3_R expression was silenced displayed significantly lower caspase-3/7 activation by Stx1 in comparison to control cells transfected with a control sequence (**Figure 2C**, for source data see **Supplementary Figure 3C**). Stx1 stimulation of control cells was defined as 100%. Lower caspase activation in cells with silenced IP_3_R supports the involvement of this receptor.

### Stx1-induced ATP release is dependent on intracellular Ca^2+^

**Figure 3:**
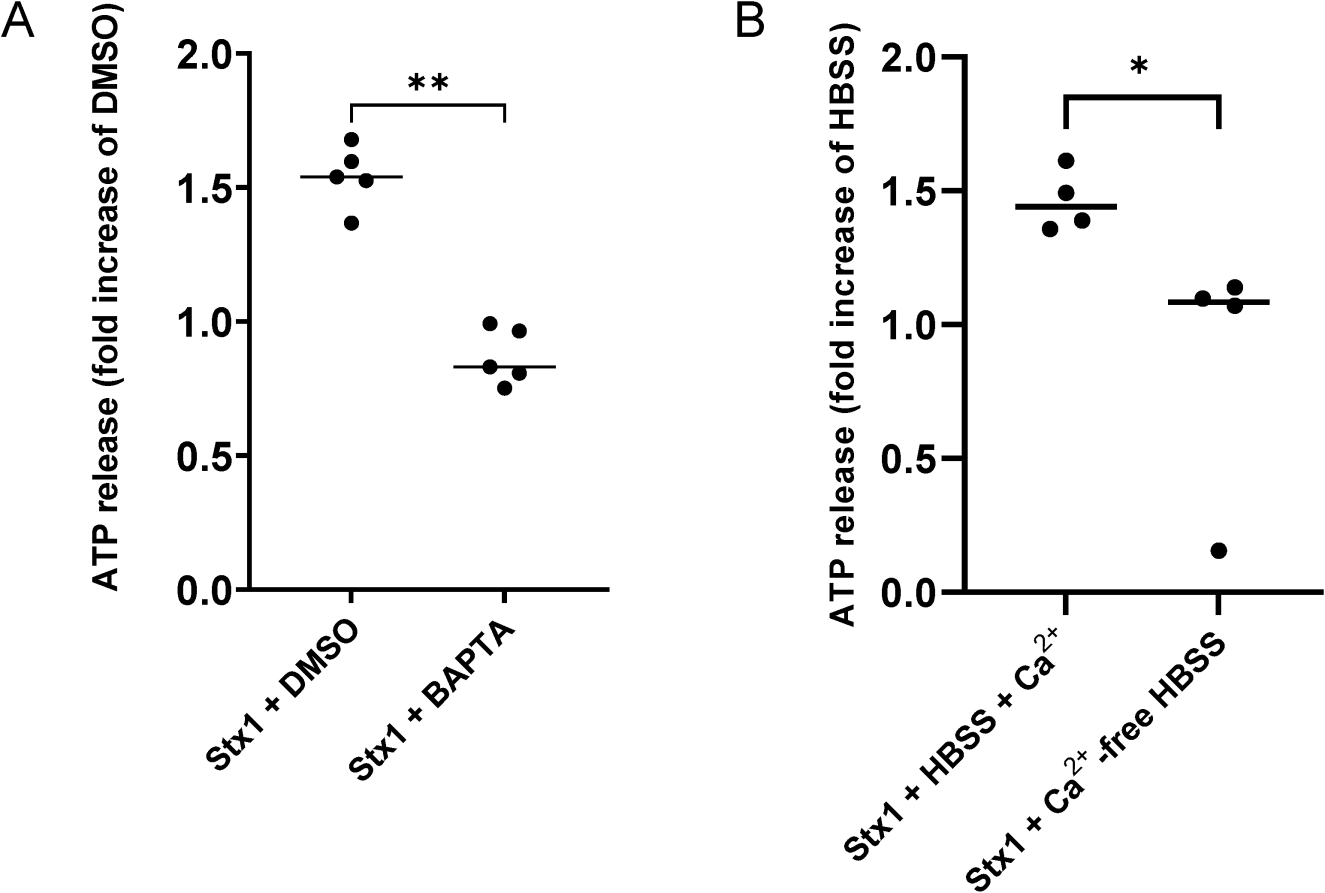
Intra- and extracellular Ca2+ is involved in Shiga toxin 1-induced ATP release **(A)** HeLa cells were incubated with BAPTA-AM, a cell permeant chelator, followed by stimulation with Shiga toxin 1 (Stx1). A significantly higher amount of ATP was found in the supernatant of Stx1-stimulated untreated cells compared to cells treated with BAPTA-AM (n=5). **(B)** HeLa cells were incubated in HBSS with and without exogenous Ca^2+^ and then stimulated with Stx1. Significantly lower amounts of ATP were detected in the supernatant of Stx1-stimulated cells that were incubated in Ca^2+^-free HBSS compared to cells in Ca^2+^-HBSS (n=4). Each data point represents an independent experiment and is a mean of 4 technical repeats. Samples were normalized to 1 based on the vehicle samples as presented in the source data (**Supplementary Figure 5**). Median ATP release is denoted by the bar. *P <0.05, **P <0.01, Mann-Whitney test.

IP_3_ binds to the IP_3_R and causes release of Ca^2+^ from the endoplasmic reticulum into the cytosol (Miyawaki et al., 1990). The intracellular Ca^2+^ chelator BAPTA-AM was used to specifically bind intracellular Ca^2+^. BAPTA-AM-treated HeLa cells stimulated with Stx1 exhibited significantly less ATP in the cell supernatant compared to DMSO-treated cells stimulated with Stx1 (**Figure 3A**, for source data see **Supplementary Figure 5**).

Lowering osmolality by addition of distilled water induces ATP release from cells (Taylor et al., 1998). HeLa cells incubated with BAPTA-AM or DMSO were therefore incubated with distilled water, as a positive control. A significantly lower amount of extracellular ATP was seen in the supernatant of cells that were incubated with distilled water followed by BAPTA-AM (**Supplementary Figure 6A**) confirming the effect of BAPTA on intracellular Ca^2+^.

### Stx1-induced ATP release is dependent on Ca^2+^ from extracellular sources

HeLa cells were incubated in HBSS with or without Ca^2+^ and stimulated with Stx1. A significantly higher amount of ATP was detected in the supernatant from the Stx1-stimulated cells exposed to extracellular Ca^2+^ compared to Stx1-stimulated cells without Ca^2+^ (**Figure 3B**). Similarly, when HeLa cells incubated in HBSS with or without Ca^2+^ were incubated in hypotonic solution with distilled water, to induce ATP release, a significantly higher amount of extracellular ATP was seen in the supernatant of the cells that were incubated in the buffer containing Ca^2+^ (**Supplementary Figure 6B**). Taken together, the results using BAPTA-AM and exogenous Ca^2+^ indicate that Stx1-mediated ATP release is dependent on both intracellular and extracellular Ca^2+^ sources.

### PIP2 staining of Stx2 injected mice

**Figure 4:**
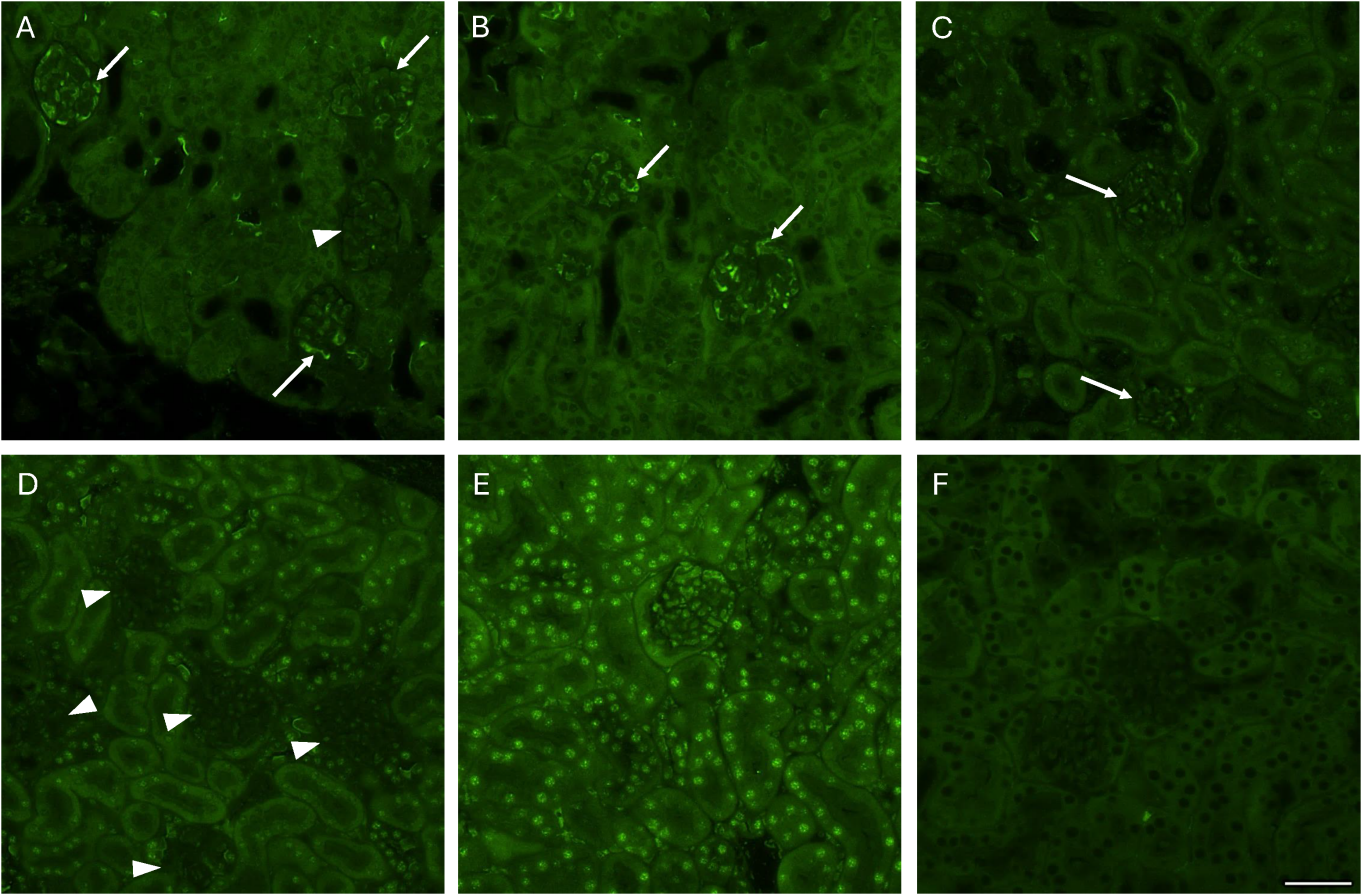
PIP2 staining in murine renal tissue Kidneys from Shiga toxin 2 (Stx2)-injected mice and controls were stained for PIP2. **(A)** Kidney from a mouse injected with Stx2 and PBS showing positive PIP2 staining in glomeruli (arrows) and one glomerulus with no staining (arrowhead). **(B)** PIP2 positive glomeruli in a mouse injected with Stx2 and DMSO. Arrows point to positive glomerular staining. **(C)** PIP2 staining in a mouse injected with Stx2 and alpelisib. Arrows point to weak glomerular staining. **(D)** PIP2 labelling in a control mouse treated with alpelisib but not with Stx2. Arrowheads point to negative glomeruli. **(E)** PIP2 labelling in a PBS control mouse showing strong nuclear staining. **(F)** Secondary antibody alone in a PBS mouse. Scale bar: 50 μm.

Mouse experiments were designed to evaluate PIP2 activation after Stx2 injection *in vivo*. Certain mice were treated with the PIK3 inhibitor alpelisib both before and after Stx2 injection. Mice were euthanized and kidneys were removed 4 days after injection. Stx2-challenged mice exhibited an increase in glomerular staining visualized in **Figures 4A, B**. Mice challenged with Stx2 and treated with alpelisib exhibited less glomerular staining (**Figure 4C**) and alpelisib alone did not show glomerular staining (**Figure 4D**). Mice that were injected with the PBS or DMSO vehicles exhibited nuclear staining (**Figure 4E**) which has been previously described (Castano et al., 2019; Sztacho et al., 2024) but did not display glomerular staining. The nuclear pattern of staining was absent in the Stx2-injected mice. The secondary antibody alone displayed no staining (**Figure 4F**). A summary of the percent of PIP2-positive glomeruli in entire kidney sections is presented in **Table 2**.

**Table 2:** Glomerular PIP2 deposits in mice.

Mouse groups	Number of mice (sex)	Positive staining in glomeruli/total number of counted glomeruli (range)	Percent PIP2-positive staining in glomeruli
Stx2 PBS	2 (1 F, 1 M)	64/150 (61-67/140-160)	43%
Stx2 DMSO	2 (1 F, 1 M)	77/160 (40-113/147-173)	48%
Stx2 Alpelisib^a^	2 (F)	36/157 (23-49/146-167)	23%
PBS control	1 (M)	2/140	1%
DMSO	1 (M)	1/218	0.5%
Alpelisib	1 (F)	1/172	0.6%

PIP2, phosphatidylinositol 4,5-bisphosphate; Stx2, Shiga toxin 2. F, female, M, male. a, treatment with alpelisib was given 1 hour before and 24 hours after Stx2.

## Discussion

Stx induces the release of ATP from cells and ATP, via purinergic P2X receptor signaling, contributes to the damaging effects of Stx such as inhibition of protein synthesis, decreased cell viability, increased apoptosis, and shedding of extracellular vesicles (Johansson et al., 2019). The presence of the Gb3 or Gb4 glycolipid receptor on cells is a prerequisite for Shiga toxin binding and induction of its cytotoxic effects (Lindberg et al., 1987; Johansson et al., 2020). Here we determined the intracellular signaling pathway associated with toxin-mediated ATP release specifically showing the involvement of the G protein alpha subunit Gi/o, of GPCRs, (blocked by Pertussis toxin), PI3K, PLC and the IP_3_ receptor. The latter was also involved in Stx1- and Stx2-induced apoptosis, as demonstrated by caspase 3/7 activation. We could show that Stx1-mediated ATP release is a calcium-dependent process that is inhibited by an intracellular Ca^2+^ chelator and by removal of extracellular Ca^2+^. The results suggest that Stx1 induces ATP release via G protein, PLC and IP_3_ signaling leading to Ca^2+^ release from the endoplasmic reticulum into the cytosol, which is required for this process.

**Figure 5:**
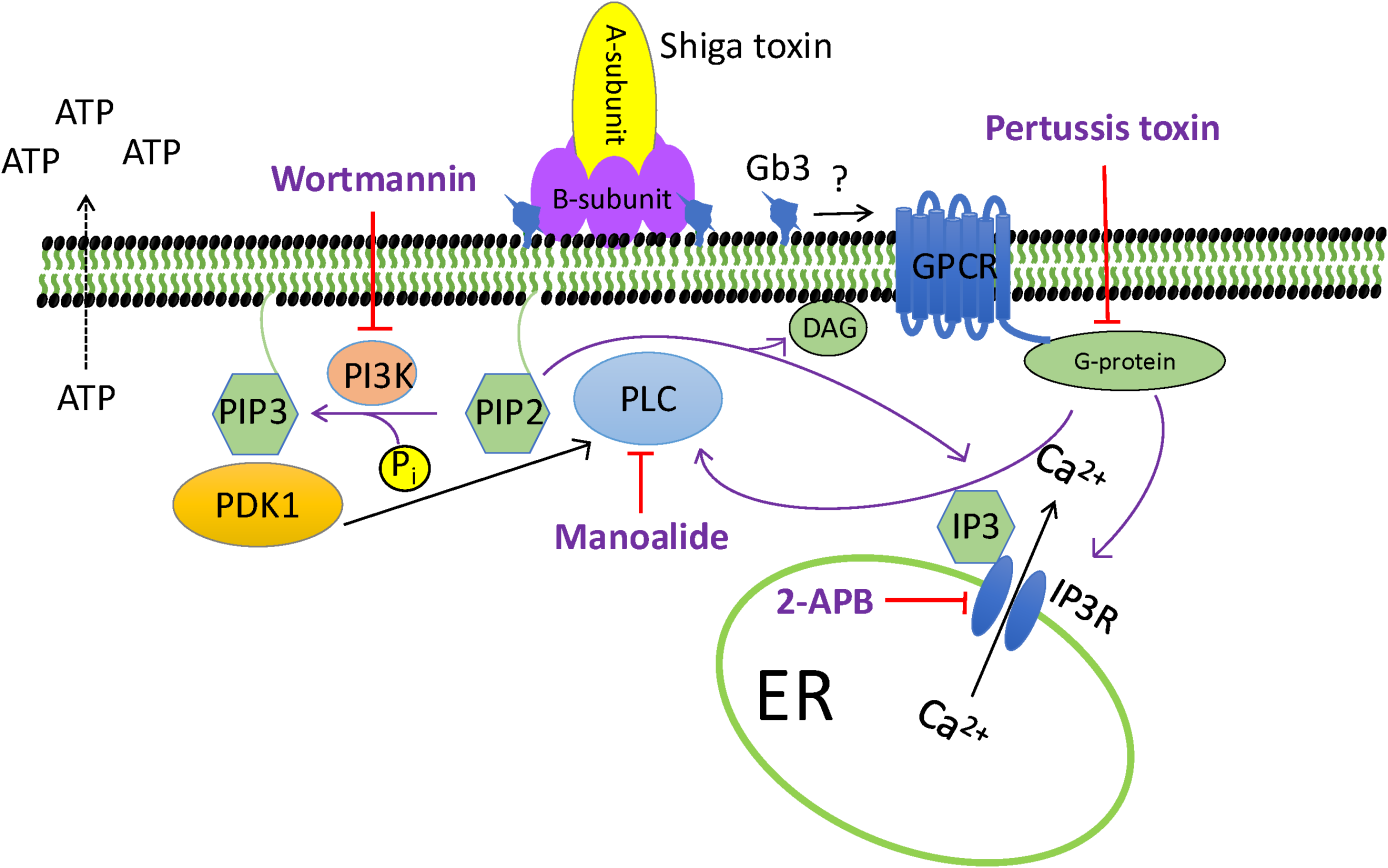
Proposed mechanism of Shiga toxin 1-mediated cellular pathways leading to ATP release A schematic presentation of the pathway inhibitors that blocked Shiga toxin 1 (Stx1)-mediated ATP release from HeLa cells in the current study. Stx1, composed of one toxic A subunit and a pentameric binding B subunit binds to the Gb3 (globotriaosylceramide) receptor. Four inhibitors were shown to have an effect on toxin-mediated ATP release: pertussis toxin, wortmannin, manoalide and 2-APB. Pertussis toxin inactivates the α-subunit of the G_i_ protein, which has been shown to induce Ca^2+^ release from the IP_3_ (inositol 1,4,5-trisphosphate) receptor. Wortmannin is a phosphoinositide 3-kinase inhibitor that blocks the phosphorylation of PIP2 (phosphatidylinositiol 4,5-bisphosphate) to PIP3 (phosphatidylinositiol 3,4,5-bisphosphate) in the plasma membrane. PIP3 binds PDK1 (3-phosphoinositide dependent protein kinase-1) which activates PLC (phospholipase C). PLC hydrolyzes PIP2 into DAG (diacylglycerol) and IP_3_. PLC is inhibited by manoalide. IP_3_ is soluble and binds to the IP_3_ receptor (IP_3_R) that is a Ca^2+^ channel on the membrane of the ER (endoplasmic reticulum), thereby leading to Ca^2+^ release from the ER into the cytosol. The IP_3_ receptor is blocked by 2-APB. ATP release is Ca^2+^ dependent. For references see the discussion.

The pathway by which toxin signaling leads to ATP release, based on the results presented in the current study, is depicted in **Figure 5**. After Stx1 binds to Gb3 signal transduction is initiated by an interaction with a GPCR and one or more Gi/oprotein. This interaction could be followed by an interaction with the phospholipid PIP2 (Yen et al., 2018) leading to phosphorylation of PIP2 to PIP3 by PI3K (Czech, 2000), which is inhibited by wortmannin, and, in the *in vivo* experiments, by alpelisib. PIP3 binds to 3-phosphoinositide dependent protein kinase-1 (PDK-1) which activates PLC (Raimondi et al., 2012; Gagliardi et al., 2015; Raimondi et al., 2017). PLC hydrolyzes PIP2 into diacylglycerol (DAG) and IP_3_ (Berridge, 2016) and its activity is inhibited by manoalide. IP_3_ binds to the IP_3_R on the membrane of the endoplasmic reticulum that is a Ca^2+^ channel and thereby leads to Ca^2+^ release from the endoplasmic reticulum into the cytosol (Berridge, 2016). The IP_3_R is blocked by 2-APB. ATP exocytosis is largely dependent on elevation of intracellular Ca^2+^ (Boudreault and Grygorczyk, 2004; Locovei et al., 2006; Xiong et al., 2018) which explains why blocking PLC, which induces IP_3_ leading to release of Ca^2+^ from the endoplasmic reticulum, lowers extracellular ATP. Pertussis-sensitive G-protein activation has been shown to activate PLC (Okajima et al., 1996) and interact with the IP_3_ receptor, as IP_3_-induced Ca^2+^ release from the ER is inhibited in the presence of Pertussis toxin (Neylon et al., 1998). G-protein activation can thereby mobilize intracellular Ca^2+^ as in the pathway depicted herein. Stx1-induced increase in intracellular calcium is decreased by a purinergic P2X1 antagonist (Johansson et al., 2019). Similarly, Liu et al. showed that Stx1 B subunit increases intracellular calcium in human umbilical vein endothelial cells (HUVEC) but Stx1 and Stx2 did not cause a rise in intracellular cAMP levels suggesting activation of a cAMP-independent signaling pathway (Liu et al., 2011).

Stx induces apoptosis of HeLa cells (Burlaka et al., 2013; Johansson et al., 2019). Depletion of Ca^2+^ in the endoplasmic reticulum mediates cell stress and, if prolonged, leads to apoptosis (Nakano et al., 2006). We demonstrated that 2-APB, blocking the IP_3_R and Ca^2+^ efflux from the endoplasmic reticulum, decreased caspase 3/7 activity in HeLa cells stimulated with Stx1 and Stx2. Thus, the protective effect of 2-APB was probably related to a decreased release of Ca^2+^ from the endoplasmic reticulum. Other studies have reported similar findings using 2-APB to inhibit apoptosis induced by cisplatin and cadmium (Splettstoesser et al., 2007; Liu et al., 2016). Importantly, we do not know if the Stx B (binding) subunit is sufficient to induce ATP release from cells, but caspase activation and Stx1-induced apoptosis are associated with the enzymatically active A subunit and, thus, the intracellular effects detected are related to the presence of the holotoxin.

In the clinical setting Stx2 is more often associated with severe EHEC infection (Freedman et al., 2023). The *in vitro* effects of Stx1 and Stx2 were supported by *in vivo* experiments injecting Stx2 i.p. into mice. Although this method of Stx2 administration does not fully replicate human infection it mimics aspects of EHEC-induced kidney damage in mice (Chromek et al., 2012). The results show, as proof of concept, that Stx2 induced enhanced glomerular PIP2 staining that was reduced by the PI3K inhibitor alpelisib. Alpelisib is a commercially available drug used for treatment of PIK3CA mutated breast cancer. The results indicate PIP2 involvement but additional experiments are required to support the role of the PIP2 pathway in Stx2-mediated signaling. A previous study showed that Stx2 released from EHEC did not activate the PIK3/Akt pathway in Gb3-negative intestinal epithelial cells (Gobert et al., 2007). These separate studies utilized differing cells (Gb3-positive versus Gb3-negative cells) and addressed different intracellular pathways. Herein we studied the PIK3-IP_3_R pathway demonstrating intracellular signaling channeled towards IP_3_R and thereby an increase in intracellular Ca^2+^.

IP_3_-mediated ATP release has been previously demonstrated by angiotensin II binding to the angiotensin 1 receptor (AT1R) on smooth muscle cells (Katsuragi et al., 2002). Similarly, binding of other agonists was shown to stimulate ATP release (Burnstock and Ralevic, 2014), for example adenosine binding to the A1 receptor on Madin-Darby canine kidney cells (Migita et al., 2005), bradykinin binding to the B2 receptor on smooth muscle cells (Zhao et al., 2010) as well as the P2Y agonist UTP and the muscarinic agonist carbachol in the urothelium (Sui et al., 2014). All these agonists utilize GPCRs which have been clearly shown to activate PLC and PI3K (Berridge, 2016). In addition to GPCRs, the receptors of tyrosine kinases have been demonstrated to play an important role in activation of PLC (Gagliardi et al., 2015).

The results suggest that Stx binding to the Gb3 receptor followed by activation of PLC signaling, leading to increased ATP release from HeLa cells, occurs by secondary activation of G protein and specifically the G_i/o_ family. The means by which Stx bound to Gb3 could activate these receptors remains to be elucidated. Stx was shown to activate the tyrosine kinase Syk (Klokk et al., 2016) which was found to be important for toxin uptake and retrograde transport (Lauvrak et al., 2006). Furthermore, tyrosine kinase inhibitors blocked the inflammatory effect of Stx on monocytic THP-1 cells (Foster et al., 2000). Infection of Hep-2 cells with STEC activated the IP_3_ pathway and increased cytosolic Ca^2+^ (Ismaili et al., 1995) although signal transduction induced by the whole bacteria may not be related to the effects of Stx alone. Of note, IP_3_-triggered Ca^2+^ efflux from the endoplasmic reticulum was shown to deplete the cell membrane of Gb3 (Malek et al., 2021) which could protect the cell from further toxic effects.

In summary, Stx1 mediates ATP release from cells and secondary cytotoxic effects via activation of Gi/o-proteins, PLC and IP_3_ signaling. This pathway is dependent on calcium from both intracellular and extracellular sources. Blockade of the IP_3_R protects HeLa cells from the damaging effects of both Stx1 and Stx2. Stx2 induced PIP2 glomerular expression in mice which was inhibited by the PIK3 inhibitor alpelisib. This study provides novel insight into a G protein signaling pathway mediated by Stx leading to ATP release and ultimately cytotoxic effects.

## Data Availability

The original contributions presented in the study are included in the article/**Supplementary Material**. Further inquiries can be directed to the corresponding author.
