# Supplementary material for: Cellular signaling pathway of Shiga toxin-induced ATP release
Source: Front Cell Infect Microbiol. 2026 Feb 23;16:1705239. doi: 10.3389/fcimb.2026.1705239 (PMC12968227; doi:10.3389/fcimb.2026.1705239)
Supplement: Supplementary file 1 [file Presentation1.pptx]

## Slide 1
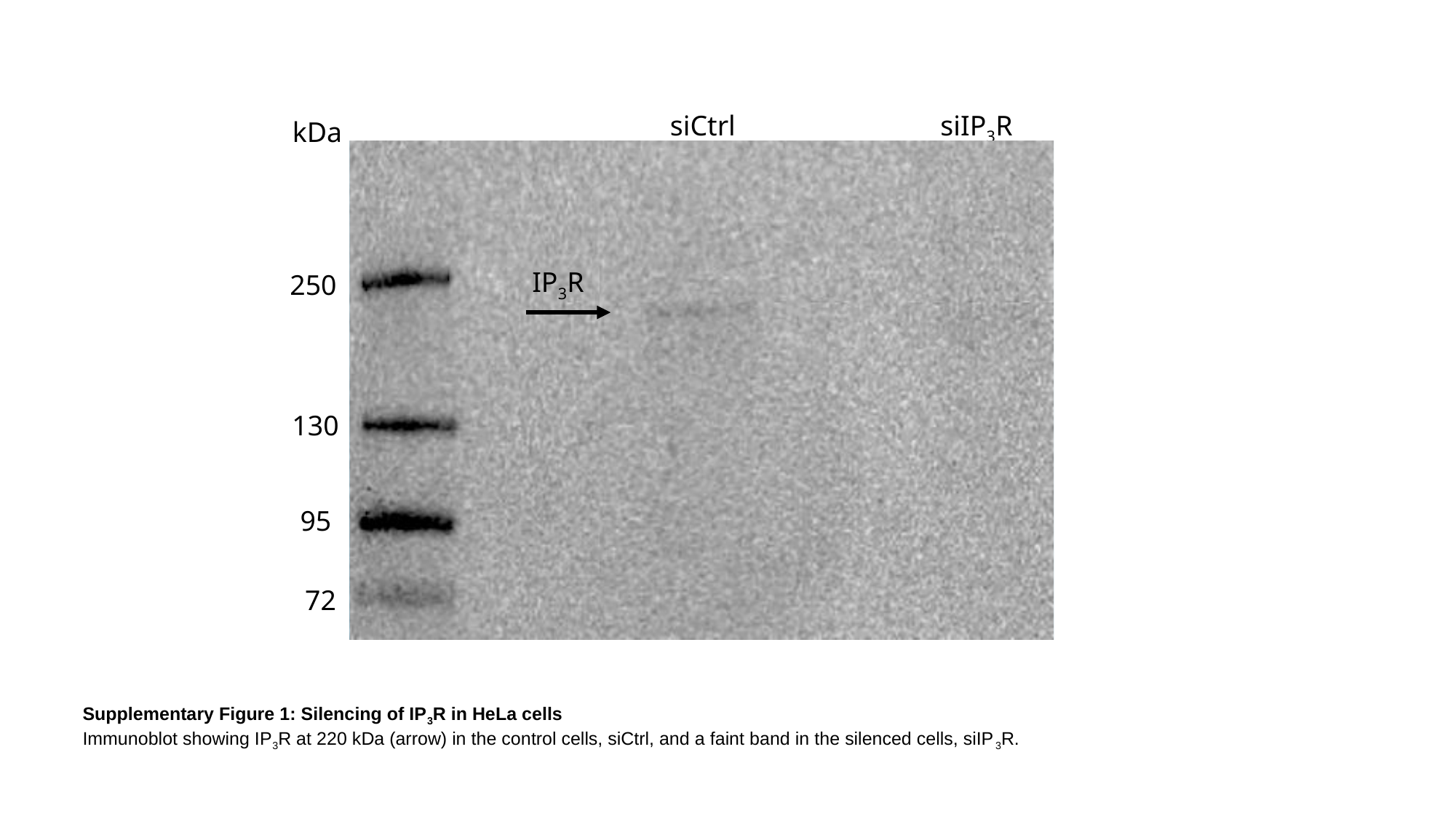

siCtrl
siIP3R
IP3R
kDa
250
130
95
72
Supplementary Figure 1: Silencing of IP3R in HeLa cells
Immunoblot showing IP3R at 220 kDa (arrow) in the control cells, siCtrl, and a faint band in the silenced cells, siIP3R.

## Slide 2
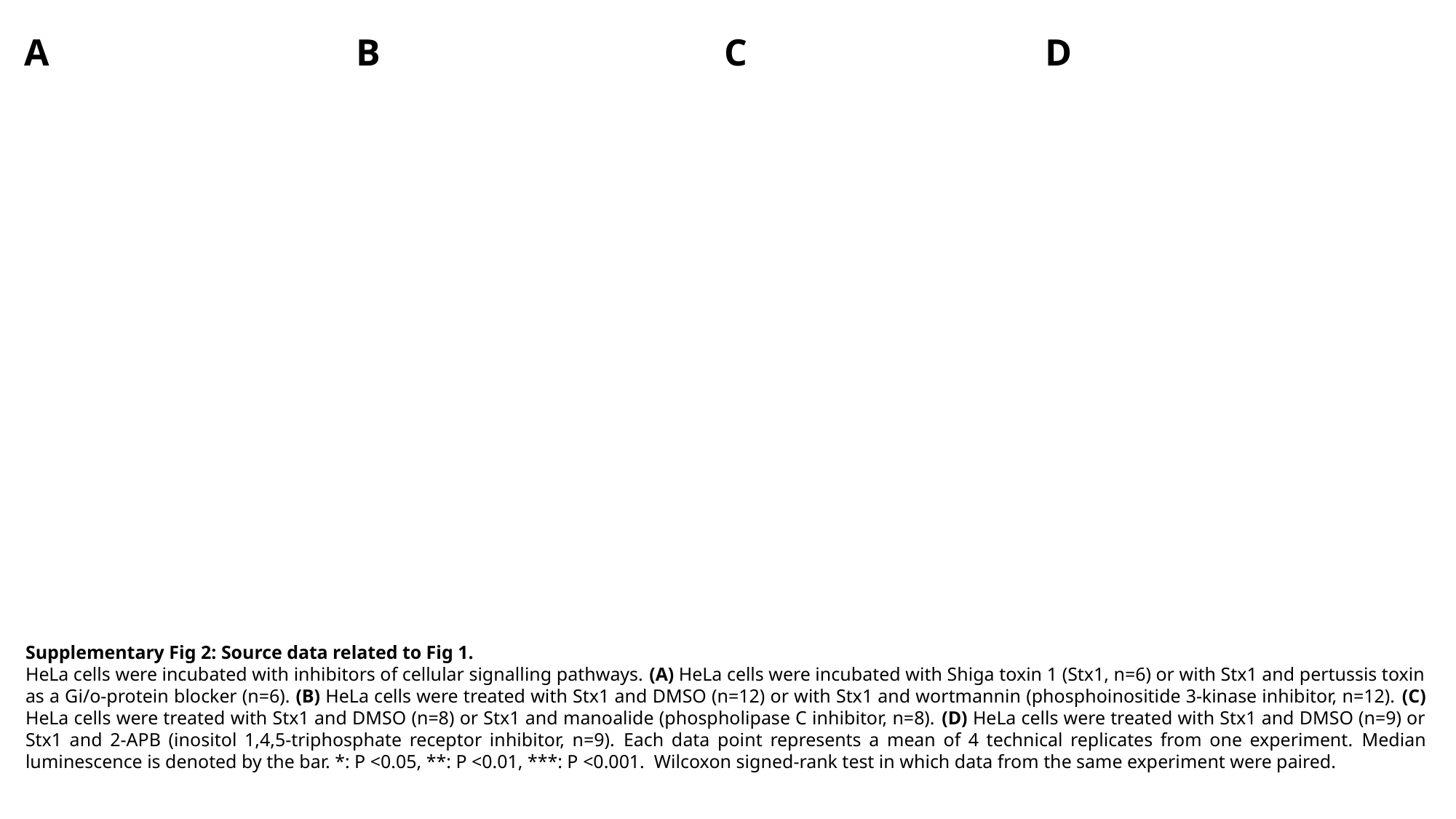

A
B
C
D
Supplementary Fig 2: Source data related to Fig 1.
HeLa cells were incubated with inhibitors of cellular signalling pathways. (A) HeLa cells were incubated with Shiga toxin 1 (Stx1, n=6) or with Stx1 and pertussis toxin as a Gi/o-protein blocker (n=6). (B) HeLa cells were treated with Stx1 and DMSO (n=12) or with Stx1 and wortmannin (phosphoinositide 3-kinase inhibitor, n=12). (C) HeLa cells were treated with Stx1 and DMSO (n=8) or Stx1 and manoalide (phospholipase C inhibitor, n=8). (D) HeLa cells were treated with Stx1 and DMSO (n=9) or Stx1 and 2-APB (inositol 1,4,5-triphosphate receptor inhibitor, n=9). Each data point represents a mean of 4 technical replicates from one experiment. Median luminescence is denoted by the bar. *: P <0.05, **: P <0.01, ***: P <0.001. Wilcoxon signed-rank test in which data from the same experiment were paired.

## Slide 3
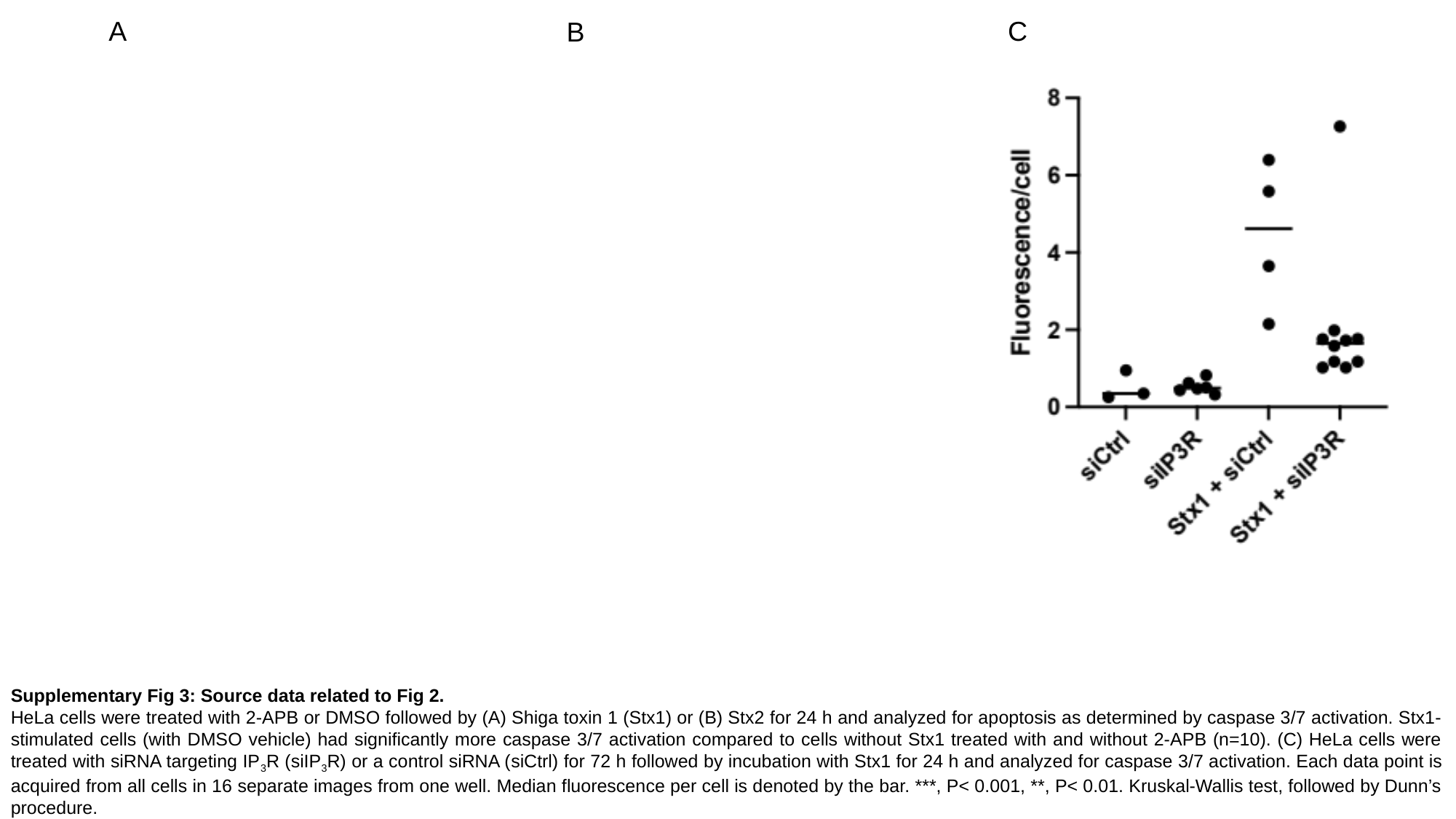

A
C
B
Supplementary Fig 3: Source data related to Fig 2.
HeLa cells were treated with 2-APB or DMSO followed by (A) Shiga toxin 1 (Stx1) or (B) Stx2 for 24 h and analyzed for apoptosis as determined by caspase 3/7 activation. Stx1-stimulated cells (with DMSO vehicle) had significantly more caspase 3/7 activation compared to cells without Stx1 treated with and without 2-APB (n=10). (C) HeLa cells were treated with siRNA targeting IP3R (siIP3R) or a control siRNA (siCtrl) for 72 h followed by incubation with Stx1 for 24 h and analyzed for caspase 3/7 activation. Each data point is acquired from all cells in 16 separate images from one well. Median fluorescence per cell is denoted by the bar. ***, P< 0.001, **, P< 0.01. Kruskal-Wallis test, followed by Dunn’s procedure.

## Slide 4
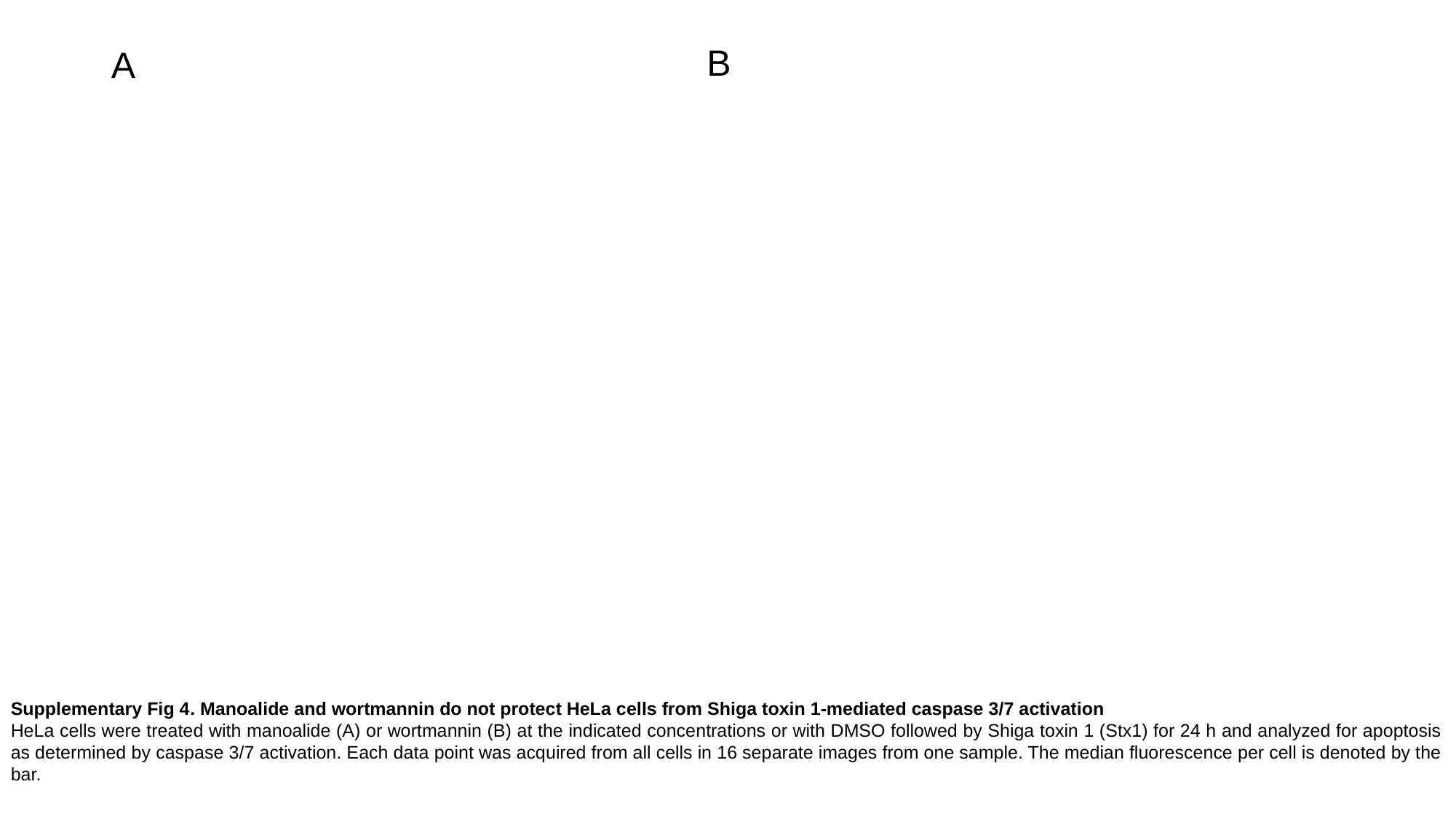

B
A
Supplementary Fig 4. Manoalide and wortmannin do not protect HeLa cells from Shiga toxin 1-mediated caspase 3/7 activation
HeLa cells were treated with manoalide (A) or wortmannin (B) at the indicated concentrations or with DMSO followed by Shiga toxin 1 (Stx1) for 24 h and analyzed for apoptosis as determined by caspase 3/7 activation. Each data point was acquired from all cells in 16 separate images from one sample. The median fluorescence per cell is denoted by the bar.

## Slide 5
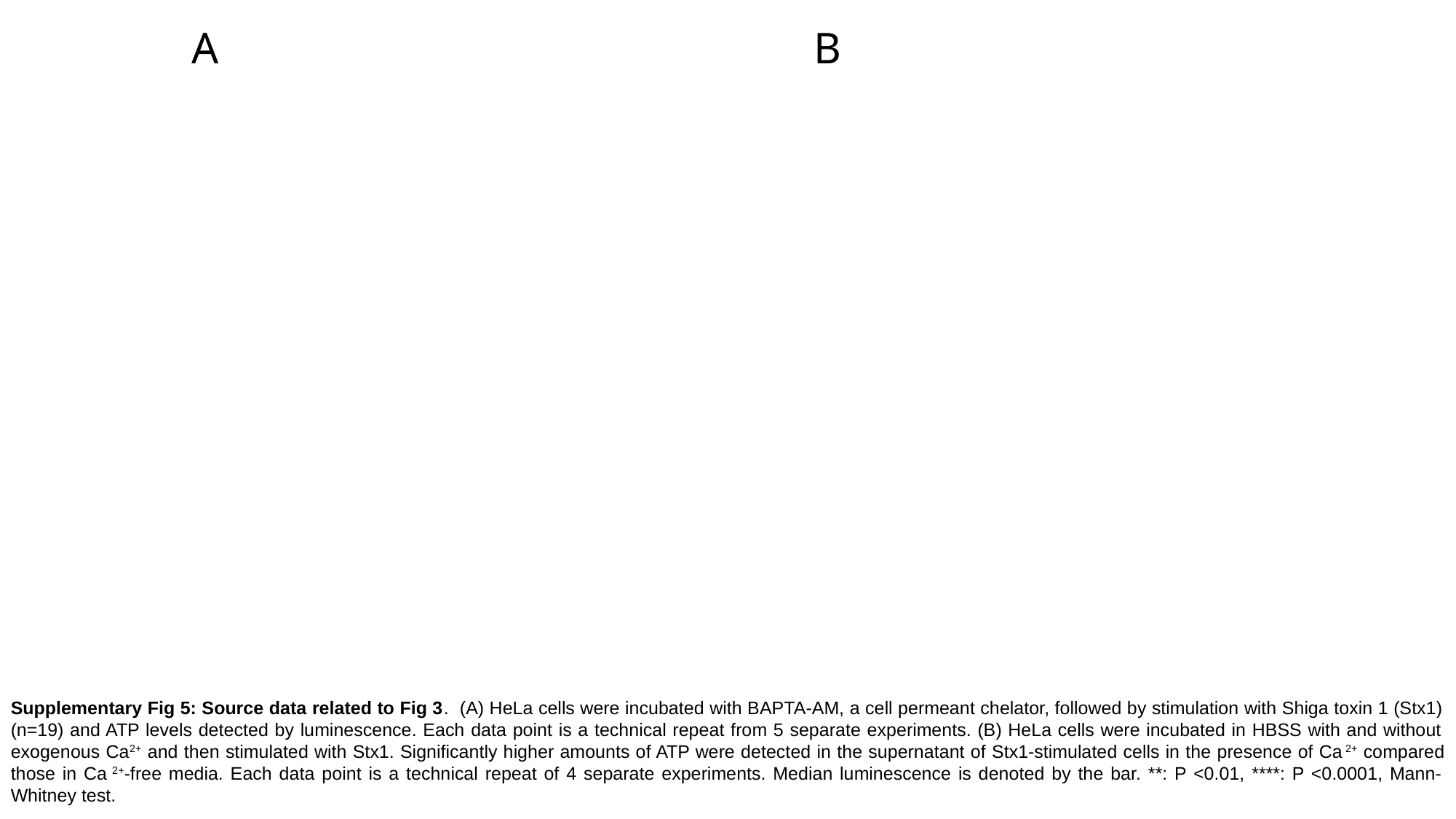

A
B
Supplementary Fig 5: Source data related to Fig 3. (A) HeLa cells were incubated with BAPTA-AM, a cell permeant chelator, followed by stimulation with Shiga toxin 1 (Stx1) (n=19) and ATP levels detected by luminescence. Each data point is a technical repeat from 5 separate experiments. (B) HeLa cells were incubated in HBSS with and without exogenous Ca2+ and then stimulated with Stx1. Significantly higher amounts of ATP were detected in the supernatant of Stx1-stimulated cells in the presence of Ca2+ compared those in Ca 2+-free media. Each data point is a technical repeat of 4 separate experiments. Median luminescence is denoted by the bar. **: P <0.01, ****: P <0.0001, Mann-Whitney test.

## Slide 6
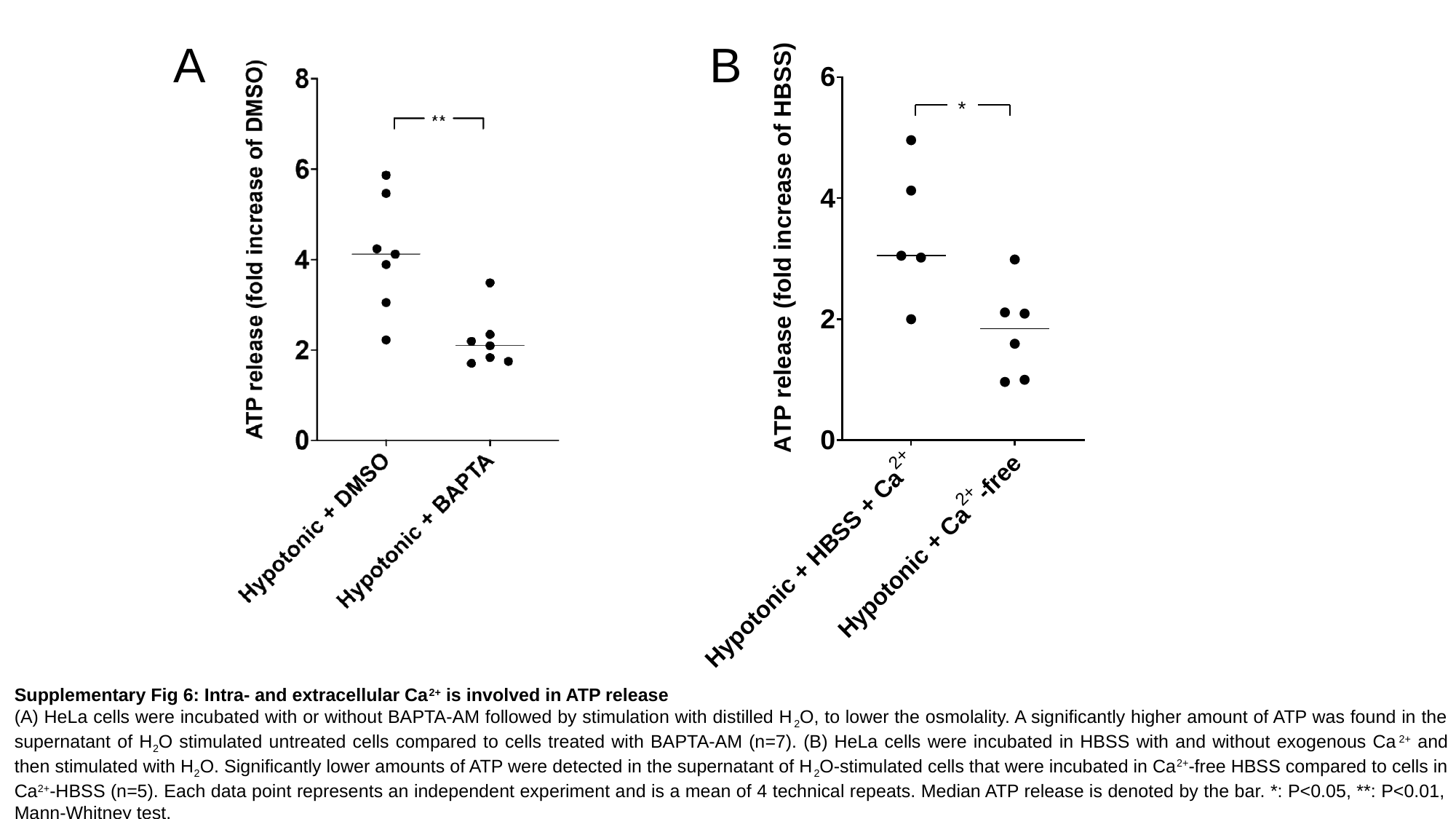

A
B
Supplementary Fig 6: Intra- and extracellular Ca2+ is involved in ATP release
(A) HeLa cells were incubated with or without BAPTA-AM followed by stimulation with distilled H2O, to lower the osmolality. A significantly higher amount of ATP was found in the supernatant of H2O stimulated untreated cells compared to cells treated with BAPTA-AM (n=7). (B) HeLa cells were incubated in HBSS with and without exogenous Ca2+ and then stimulated with H2O. Significantly lower amounts of ATP were detected in the supernatant of H2O-stimulated cells that were incubated in Ca2+-free HBSS compared to cells in Ca2+-HBSS (n=5). Each data point represents an independent experiment and is a mean of 4 technical repeats. Median ATP release is denoted by the bar. *: P<0.05, **: P<0.01, Mann-Whitney test.
